# Mass Spectrometric Analysis of Purine Intermediary Metabolism Indicates Cyanide Induces Purine Catabolism in Rabbits

**DOI:** 10.3390/metabo14050279

**Published:** 2024-05-10

**Authors:** Jordan Morningstar, Jangwoen Lee, Sari Mahon, Matthew Brenner, Anjali K. Nath

**Affiliations:** 1 Division of Cardiovascular Medicine, Beth Israel Deaconess Medical Center, Boston, MA 02215, USA; 2 Beckman Laser Institute, University of California, Irvine, CA 92697, USAmahonsb@hs.uci.edu (S.M.); mbrenner@uci.edu (M.B.); 3 Division of Pulmonary and Critical Care Medicine, Department of Medicine, University of California, Irvine, CA 92697, USA; 4 Harvard Medical School, Boston, MA 02215, USA

**Keywords:** cyanide, mass spectrometry, purine, uric acid, nucleoside/nucleotide metabolism, cytochrome c oxidase (Complex IV), preclinical animal model, allopurinol, histotoxic hypoxia, biomarker

## Abstract

Purines are the building blocks of DNA/RNA, energy substrates, and cofactors. Purine metabolites, including ATP, GTP, NADH, and coenzyme A, are essential molecules in diverse biological processes such as energy metabolism, signal transduction, and enzyme activity. When purine levels increase, excess purines are either recycled to synthesize purine metabolites or catabolized to the end product uric acid. Purine catabolism increases during states of low oxygen tension (hypoxia and ischemia), but this metabolic pathway is incompletely understood in the context of histotoxic hypoxia (i.e., inhibition of oxygen utilization despite normal oxygen tension). In rabbits exposed to cyanide—a classical histotoxic hypoxia agent—we demonstrated significant increases in several concordant metabolites in the purine catabolic pathway (including plasma levels of uric acid, xanthosine, xanthine, hypoxanthine, and inosine) via mass spectrometry-based metabolite profiling. Pharmacological inhibition of the purine catabolic pathway with oxypurinol mitigated the deleterious effects of cyanide on skeletal muscle cytochrome c oxidase redox state, measured by non-invasive diffuse optical spectroscopy. Finally, plasma uric acid levels correlated strongly with those of lactic acid, an established clinical biomarker of cyanide exposure, in addition to a tissue biomarker of cyanide exposure (skeletal muscle cytochrome c oxidase redox state). Cumulatively, these findings not only shed light on the in vivo role(s) of cyanide but also have implications in the field of medical countermeasure (MCM) development.

## 1. Introduction

Cyanide is a primordial chemical reductant that may have played a role in the origins of protobiotic carbon metabolism [1]. It has also recently been discovered to be a gaseous signaling molecule in mammals [2]. However, its most well-known role is as a poison. Cyanide is a rapid-acting asphyxiant that blocks cells from utilizing oxygen. Its anions are highly reactive nucleophiles that inhibit the catalytic activity of cytochrome c oxidase thereby preventing electron transfer to O_2_ and the production of ATP. The inhibitory effect of cyanide on mitochondrial respiration is well-established and is its canonical mechanism of toxicity [3,4,5,6]. However, cyanide also binds to metal atoms in other metalloenzymes, in addition, it reacts with the carbonyl groups of organic compounds and the cysteine residues of proteins (S-cyanylation) [7,8,9,10,11,12,13]. Thus, beyond its detrimental effects on oxidative phosphorylation, cyanide disrupts numerous other biological and metabolic pathways, many of which are less well-characterized.

Combining mass spectrometry-based metabolite profiling with in vivo models of cyanide exposure, we have begun to characterize the metabolite disturbances downstream of cyanide exposure [14,15]. In rabbits exposed to sublethal cyanide and a pilot study of humans treated with nitroprusside, we have previously detected an increase in three purine metabolites in the circulation [14]. Specifically, plasma levels of inosine, hypoxanthine, and uric acid were increased during exposure to a nonlethal dose of cyanide. These three metabolites constitute part of the purine degradation pathway. This catabolic pathway breaks down purine nucleotides and nucleosides including adenosine monophosphate (AMP), guanosine monophosphate (GMP), xanthosine monophosphate (XMP), adenosine, guanosine, and xanthosine [16]. Ultimately, xanthine oxidoreductase (XOR) generates the end product of purine catabolism, i.e., uric acid, which is excreted in urine. The observation that uric acid increased was surprising because cyanide has been shown to inhibit the activity of XOR in vitro via extraction of sulfur atoms or binding iron atoms in the enzyme [17,18,19,20]. Thus, these enzyme activity studies with XOR in vitro suggest that in vivo purine catabolism to uric acid should also be inhibited by cyanide. 

Here, we sought to further interrogate the effects of cyanide on purine catabolism in the context of a living organism. To extend our initial investigations that had been made in the sublethal rabbit model, in the present study, a preclinical cyanide model was used. In our preclinical model, rabbits were exposed to a lethal infusion of cyanide, and purine metabolites were measured in serial blood samples. In addition, in this model, non-invasive diffuse optical spectroscopy is used to quantify skeletal muscle tissue cytochrome c oxidases redox state in real-time. In our prior study using sublethal cyanide, control sham animals were not assessed. In this study, we compared rabbits exposed to a lethal dose of cyanide to sham animals (anesthetized, instrumented, and injected with saline instead of cyanide). Changes in additional purine metabolites not previously found in the rabbit sublethal cyanide model were identified here in the preclinical model. Next, to assess the functional significance of these metabolite findings, we pharmacologically inhibited XOR activity in zebrafish and rabbits to determine the effects on purine metabolite levels, cytochrome c oxidase state, and survival in the context of cyanide poisoning. Finally, to assess whether uric acid could serve as a biomarker for cyanide, we determined whether uric acid levels correlate with those of an established clinical biomarker of cyanide (lactic acid). Collectively, these results provide evidence that cyanide induces purine catabolism to uric acid in vivo and that inhibition of XOR abrogates cyanide-induced inhibition of cytochrome c oxidase; in addition, uric acid may serve as a biomarker of cyanide exposure, and cyanide’s effect on cytochrome c oxidase in preclinical studies.

## 2. Experimental Procedures

### 2.1. Rabbits

All protocols and procedures were approved by the Institutional Animal Care and Use Committee (IACUC) at the University of California Irvine and comply with federal and state regulations for the protection of animals (ethical approval code: AUP-21-112; approval date: 10 July 2021). New Zealand White rabbits (female, 4 kg, 4–6 months old; Western Oregon Rabbit Supply) were anesthetized with ketamine and xylazine, intubated, and ventilated. Femoral artery and vein access were obtained to collect blood samples and measure systemic pressure. A lethal cyanide dose was achieved by continuous intravenous administration of 20 mg sodium cyanide in 60 mL of saline at a rate of 1 cc/min (0.33 mg/min). Rabbits were monitored for cyanide poisoning in real-time using standard hemodynamics, gas exchange measures, and optical technologies including continuous wave near-infrared spectroscopy and diffuse optical spectroscopy (see below). Baseline and serial blood samples were collected at the indicated time points in EDTA tubes and centrifuged. The plasma was flash-frozen and stored at −80 °C for mass spectrometry analysis.

### 2.2. Diffuse Optical Spectroscopy (DOS)

Our DOS platform enables noninvasive quantitation of tissue cytochrome c oxidase redox state via optical technology [21,22,23,24,25,26]. A fiber optic probe with a light diode emitter and detector was placed on the shaved surface of the right inner thigh and measurements are acquired continuously every 36 s. Our platform uses multifrequency domain photon migration (FDPM) with time-independent near-infrared (NIR) spectroscopy to measure bulk tissue absorption and scattering spectra. It uses five laser diodes at discrete wavelengths (661, 681, 783, 823, and 850 nm) and a fiber-coupled avalanche photodiode (APD) detector for the frequency domain measurements. The APD detects the intensity-modulated diffuse reflectance signal at modulation frequencies between 50 and 300 MHz after propagation through the tissue. Absorption and reduced scattering coefficients are measured directly at each of the five laser diode wavelengths using frequency-dependent phase and amplitude data. Steady-state acquisition is accomplished using a broadband reflectance measurement from 650 to 1000 nm that follows frequency domain measurements using a tungsten-halogen light source and a spectrometer. Intensity of the steady-state reflectance measurements is calibrated to the frequency domain values of absorption and scattering to establish the absolute reflectance intensity. To calculate cytochrome c oxidase redox state, reduced scattering coefficients are calculated as a function of wavelength throughout the near-infrared region by fitting a power-law to five reduced scattering coefficients at the baseline and are set as constant values in subsequent measurements. The change in cytochrome c oxidase redox state (ΔCcOrdx) is the change from baseline of the differential between oxidized and reduced cytochrome c oxidase concentrations. It is calculated by linear least squares fit of the wavelength-dependent extinction coefficient spectra. We used difference extinction spectra of cytochrome c oxidase [27] for the subsequent fitting and analysis.

### 2.3. Oxypurinol Administration

Oxypurinol was administered IV at a dose of 12.5 mg/kg. Oxypurinol (50 mg) was dissolved in 1 mL of 1M NaOH by briefly heating (95 °C) and subsequently vortexing. Next, 8 mL of 0.9% saline was added, followed by 250 µL of 1M HCl to neutralize pH. The drug was administered 5 min prior to the start of the cyanide infusion. 

### 2.4. Glyoxylate and Hexachloroplaninate Administration

Plasma samples were obtained from rabbits intramuscularly treated with glyoxylate (50 mg/kg) and hexachloroplatinate (30 mg/kg), as previously described [24,28]. These doses of antidote yield 100% survival following cyanide exposure at its LD_100_ [24,28].

### 2.5. Targeted Metabolomics

Glycolytic, TCA cycle, and purine metabolites were detected by LC-MS/MS using multiple reaction monitoring (MRM). Metabolites were measured in 30 µL of deproteinized rabbit plasma. Proteins were removed using a 75:25 *v*/*v* acetonitrile:methanol solution that was spiked with isotopically labeled and deuterated internal standards (10 μM inosine-^15^N_4_, 25 μM thymine-d_4_, 10 μM citrulline-d_7_, and 25 μM phenylalanine-d_8_). The samples were separated using a 2.1 × 100 mm 3.5-μm Xbridge amide column (Waters). Mobile phase A was 95:5 (*v*/*v*) water/acetonitrile, with 20 mM ammonium acetate and 20 mM ammonium hydroxide (pH 9.5). Mobile phase B was acetonitrile. In amide-negative mode, the chromatography system consisted of a 1260 Infinity autosampler (Agilent, Lexington, MA, USA) connected to a 1290 Infinity HPLC binary pump system (Agilent). The eluents were detected in negative mode on a coupled 6490 QQQ mass spectrometer equipped with an electrospray ionization source. The settings were as follows: sheath gas temperature, 400 °C; sheath gas flow, 12 l/min; drying gas temperature, 290 °C; drying gas flow, 15 l/min; capillary, 4000 V; nozzle pressure, 30 psi; nozzle voltage, 500 V; and delta EMV, 200 V [29]. MRM transitions for each compound (Table S1) were assessed for sensitivity, selectivity, and retention time in the pooled plasma matrix with and without the spiked reference standard. LC-MS data were quantified using Agilent MassHunter Quantitative Analysis software (Version 10). Blinded to experimental treatment and time points, an observer manually reviewed metabolite peaks for peak quality and compared them against a known standard to confirm identities. A quality control pooled plasma sample was interspersed throughout the run at regular intervals to monitor the temporal drift in mass spectrometry performance and assess the variability of each metabolite. 

### 2.6. Pharmacokinetics of Oxypurinol

MRM transitions for oxypurinol were optimized (Table S1). To generate the oxypurinol standard curve, oxypurinol was spiked into pooled reference plasma, serially diluted, and analyzed by LC-MS. The standard curve was linear from 122 nM to 31.25 µM (Figure S3). The absolute concentration of oxypurinol in treated animals was calculated using the standard curve.

### 2.7. Zebrafish

Animals were maintained and embryos were obtained according to standard fish husbandry protocols that were approved by the BIDMC IACUC and are in compliance with federal and state regulations (ethical approval code: 047-2020-23; approval date: 5 November 2023). Zebrafish embryos (Ekkwill strain) were grown in Tübingen E3 medium at 28 °C within light/dark cycle incubators. The cyanide assay was conducted on 6-day post-fertilization (d.p.f.) larvae loaded into the wells of 96-well plates containing HEPES-buffered Tübingen E3 medium (*n* = 5 larvae per well). Potassium cyanide was added, and the plates were sealed using adhesive PCR plate seals. A dose of 25 µM potassium cyanide at 28 °C induces 100% death of 6 d.p.f. larvae within 3 h. Compounds were screened in this assay using a 12-point dose–response curve of 0.4–800 µM. Viability was assessed by observing heart rate and touch response. 

### 2.8. Statistics

Analyses were performed in GraphPad Prism version 9. Metabolite levels were normalized to the baseline (pre-cyanide) peak area on a metabolite-by-metabolite basis. Data are displayed as the mean of the fold change and standard error of the mean. Significance was assessed using Mann–Whitney tests. To adjust for multiple hypothesis testing, we applied the Benjamini, Krieger, and Yekutieli 2-stage step-up method for false discovery rate (FDR) adjustment with a threshold for a statistical significance of *q* < 0.05.

## 3. Results

### 3.1. Plasma Levels of Purine Metabolites Increase in Rabbits Exposed to a Lethal Dose of Cyanide

Rabbits were anesthetized, instrumented, and continuously infused (rate = 0.33 mg/min IV for 60 min) with a dose of sodium cyanide that is lethal (i.e., 20 mg total dose). Blood samples were collected before the start of the cyanide infusion (baseline) and at 5, 15, 25, 40, and 55 min into the cyanide infusion. To identify potential confounding metabolite changes that are independent of the cyanide’s effect and reflect instrumentation or anesthesia effects, sham animals were infused with saline rather than cyanide. 

First, we confirmed that the cyanide perturbation in this cohort induced expected derangements in the known cyanide-sensitive metabolic pathways. As tissues shift from oxidative phosphorylation to glycolysis in cyanide-poisoned rabbits, we previously observed an accumulation of circulating glycolytic and TCA cycle metabolites measured by liquid chromatography-tandem mass spectrometry (LC-MS/MS) [24,28,29]. As expected, rabbits infused with a lethal dose of cyanide exhibited significantly elevated levels of lactic acid (FDR *q* = 0.001, *n* = 15) and pyruvic acid (FDR *q* = 0.00002, *n* = 15) in addition to elevated levels of TCA metabolites (oxaloacetic acid, aconitic acid, alpha-ketoglutaric acid, succinic acid, fumaric acid, and malic acid; FDR *q* = 0.00002, *n* = 15, Table 1 and Figure S1A–H). In sham controls, the levels of circulating glycolytic and TCA cycle metabolites remained near the baseline throughout the study (*n* = 5, Table 1 and Figure S1A–H).

**Figure 1:**
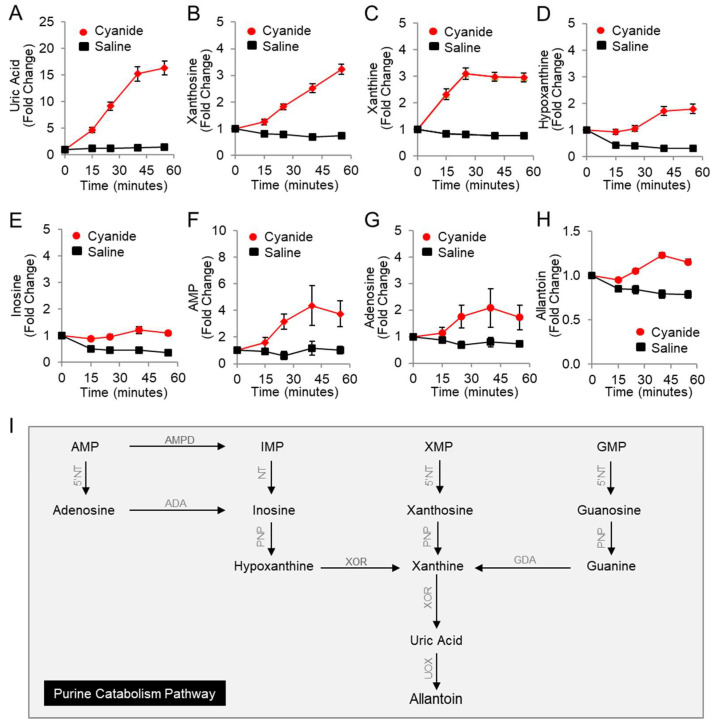
Plasma levels of purine metabolites increase in rabbits exposed to a lethal dose of cyanide. Metabolites were measured in the plasma of cyanide-treated animals (*n* = 15) and sham control animals that received saline instead of cyanide (*n* = 5): (**A**) uric acid; (**B**) xanthosine; (**C**) xanthine; (**D**) hypoxanthine; (**E**) inosine; (**F**) AMP; (**G**) adenosine; and (**H**) allantoin. Each metabolite is normalized to its baseline value (t = 0), i.e., before infusion with cyanide or saline. Data are presented as the mean ± SEM. See Table 2 for *p* values and *q* values. (**I**) Diagram of the purine catabolism pathway. 5′-Nucleotidase (5′NT) removes the phosphate group from nucleotides (AMP, IMP, XMP, and GMP) and generates nucleosides (adenosine, inosine, xanthosine, and guanosine). Purine nucleoside phosphorylase (PNP) converts inosine, xanthosine, and guanosine to hypoxanthine, xanthine, and guanine. Xanthine oxidoreductase (XOR) ultimately generates the end product in purine metabolism, uric acid. In rabbits, but not humans, urate oxidase (UOX) converts uric acid to allantoin. AMPD = adenosine monophosphate deaminase, ADA = adenosine deaminase, and GDA = guanine deaminase.

Using LC-MS, eight purine metabolites were measured in the plasma of rabbits (Table 2 and Figure 1A–I) including nucleotides, nucleosides, nucleobases, and end products of purine catabolism (uric acid and allantoin). Uric acid significantly increased in cyanide-treated animals as compared to saline-treated animals by 13-fold (FDR *q* = 0.0003, Figure 1A), hypoxanthine increased 5-fold (FDR *q* = 0.0005; Figure 1D), and inosine increased 2.6-fold (FDR *q* = 0.001; Figure 1E). Moreover, significant increases in additional purine metabolites were observed in response to cyanide treatment (Table 2): xanthosine (4.6-fold, *q* = 0.0003, Figure 1B), xanthine (4-fold, *q* = 0.0003, Figure 1C), AMP (3-fold, *q* = 0.0286, Figure 1F), and allantoin (1.6-fold, *q* = 0.0003, Figure 1H). Together, these concordant metabolic findings indicate that cyanide induces purine catabolism to uric acid in rabbits (Figure 1I). In addition, the formation of uric acid suggests that xanthine oxidoreductase, the rate-limiting enzyme in uric acid production, can function in the milieu of cyanide in vivo.

### 3.2. Pharmacological Inhibition of Purine Catabolism Confers Protection against Cyanide Poisoning in Zebrafish

We next sought to determine the effects of inhibiting purine catabolism in the context of cyanide. Specifically, we tested the hypothesis that blocking the rate-limiting enzyme in purine catabolism (xanthine oxidoreductase) confers a survival advantage in the context of cyanide. To accomplish this, an established small-molecule screening assay was conducted in cyanide-poisoned zebrafish larvae [14,23]. In this assay, zebrafish larvae are exposed to a dose of potassium cyanide (25 µM) that induces 100% lethality within 3 h. To confirm that this cyanide perturbation in zebrafish larvae induced derangements in glycolytic and TCA cycle metabolites, metabolites were measured in lysates from larvae exposed to 25 µM cyanide for 2 h (Figure S2).

**Figure 2:**
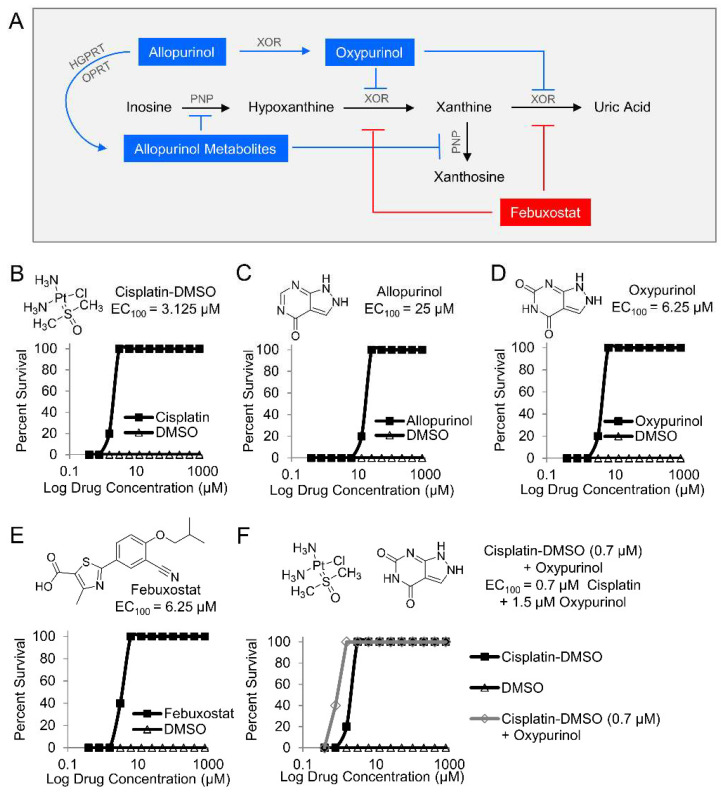
Pharmacological inhibition of purine catabolism confers protection against cyanide poisoning in zebrafish. (**A**) Allopurinol is metabolized by hypoxanthine-guanine phosphoribosyltransferase (HGPRT), the enzyme that converts guanine to guanine monophosphate (GMP), and orotate phosphoribosyltransferase (OPRT), the enzyme that converts orotate to orotate monophosphate (OMP). The allopurinol metabolites generated from those reactions inhibit purine nucleoside phosphorylase (PNP) from its activities in purine catabolic reactions. Allopurinol is also metabolized by xanthine oxidoreductase (XOR) into oxypurinol which binds to XOR and inhibits its further enzymatic activity. In contrast to allopurinol, oxypurinol and fenbuxostat are specific inhibitors of XOR. Effect of compounds on survival of zebrafish larvae exposed to a lethal dose of cyanide *(n* = 5 per well, 3 replicates) and treated with (**B**) cisplatin-DMSO (positive control); (**C**) allopurinol; (**D**) oxypurinol; (**E**) febuxostat; and (**F**) combination of subeffective dose of cisplatin-DMSO and increasing doses of oxypurinol.

Next, two purine analogs (allopurinol and oxypurinol) and a structurally distinct inhibitor (febuxostat) were tested in zebrafish. Allopurinol inhibits xanthine oxidoreductase and purine nucleoside phosphorylase (PNP), while oxypurinol and febuxostat inhibit xanthine oxidoreductase (Figure 2A). Zebrafish larvae were treated with the indicated inhibitors in a 12-point dose–response curve (0.4–800 µM) and exposed to 25 µM potassium cyanide. A known cyanide antidote (cisplatin-DMSO), which acts by chelating cyanide, was used as a positive control (EC_100_ = 3.125 µM, Figure 2B) [23]. Treatment allopurinol, oxypurinol, and febuxostat prevented cyanide-induced death in zebrafish larvae (EC_100_ = 25 µM, EC_100_ = 6.25 µM, and EC_100_ = 6.25 µM, respectively, Figure 2C–E, *n* = 3). Further, co-treatment of 0.7 µM oxypurinol plus a cyanide-chelating drug (cisplatin-DMSO) lowered the EC_100_ of cisplatin-DMSO to 1.5 µM (Figure 2F, *n* = 3). These complementary findings suggest that purine catabolism plays a role in the mechanism of cyanide toxicity either directly through xanthine oxidoreductase or indirectly by redirecting purine metabolites to other metabolic pathways.

### 3.3. Oxypurinol Abrogates the Deleterious Effects of Cyanide on Cytochrome c Oxidase in Rabbits Exposed to a Lethal Dose of Cyanide 

**Figure 3:**
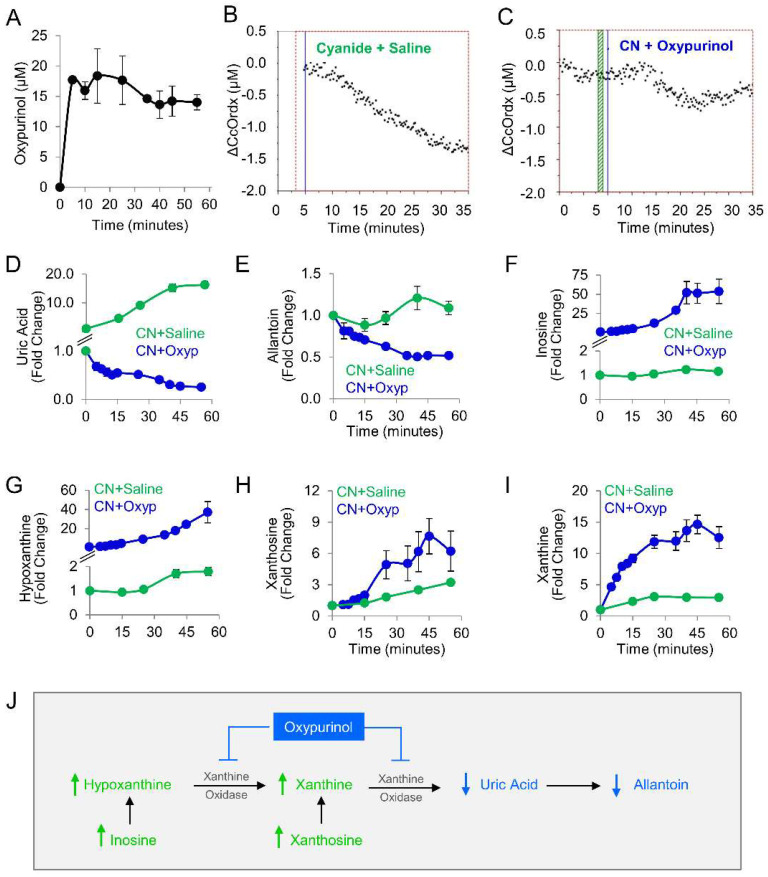
Oxypurinol abrogates the deleterious effects of cyanide on cytochrome c oxidase in rabbits exposed to cyanide. (**A**) Pharmacokinetic profile of oxypurinol in rabbits. Cytochrome c oxidase redox state (ΔCcOrdx) in the skeletal muscle of cyanide-exposed rabbits treated with (**B**) saline (9 mL IV; *n* = 9) or (**C**) oxypurinol (50 mg/kg IV; *n* = 4); horizontal lines denote the start of cyanide and treatment. Metabolites were measured in the plasma of cyanide-treated animals that received saline (*n* = 12) or oxypurinol (*n* = 4): (**D**) uric acid; (**E**) allantoin; (**F**) inosine; (**G**) hypoxanthine; (**H**) xanthosine; and (**I**) xanthine (see Table 3 for *p* values and *q* values). (**J**) Depiction of the expected effects of oxpurinol administration in this model. ↑ denotes increased levels; ↓ denotes decreased levels.

Given our findings in the zebrafish model, oxypurinol was next tested in rabbits. First, a pharmacokinetic study was performed in rabbits that received 12.5 mg/kg oxypurinol (50 mg total dose). This dose was the maximum amount of drug that could be injected based on the solubility limits of oxypurinol and injection volume limits in our IACUC protocol. A mass spectrometry-based analytical method was developed to quantify absolute concentrations of oxypurinol in rabbit plasma (Figure S3). At 5 min post-injection, 17.71 ± 0.50 µM oxypurinol was detected in the plasma. Oxypurinol concentration remained steady until 35 min post-injection when its values decreased by 18% and plateaued at 14.61 ± 0.01 µM (Figure 3A).

We used a well-established rabbit model of cyanide poisoning in which cytochrome c oxidase redox state in tissues can be monitored non-invasively and in real-time via diffuse optical spectroscopy (DOS) from a fiber optic probe placed on the shaved surface of the inner thigh [21,22,23,24,25,26]. During the progressive cyanide infusion (rate = 0.33 mg/min IV, 20 mg total dose), the cellular cytochrome c oxidase redox state (ΔCcOrdx) continuously decreased in rabbit skeletal muscle (Figure 3B, *n* = 9). This is consistent with a shift in redox state from reduced to oxidized in the heme a_3_-Cu_B_ binuclear center of cytochrome c oxidase. By contrast, in animals treated with oxypurinol (12.5 mg/kg IV) and infused with cyanide 5 min later, cyanide’s effect on cytochrome c oxidase redox state (ΔCcOrdx) was abrogated. Initially, the redox state decreased during the first 15 min of cyanide infusion, similar to the control group, but then, it plateaued at ΔCcOrdx = −0.5 µM and subsequently began to return towards baseline (Figure 3C, *n* = 4). However, in control animals that received saline followed by cyanide, the redox state continued to decrease (Figure 3B). There was a significant improvement in ΔCcOrdx in oxypurinol-treated rabbits compared to saline-treated rabbits (0.41 ± 0.21 versus 0.73 ± 0.09, *p* = 2 × 10^−5^ at 30 min into the cyanide infusion. These findings indicated that oxypurinol treatment mitigates the deleterious effects of cyanide on the redox state of cytochrome c oxidase in vivo.

At the dose used in this study (12.5 mg/kg), oxypurinol-mediated abrogation of cyanide effects on cytochrome oxidase c was not sufficient for survival. All animals died (*n* = 4), survival being considered as surviving 30 min past the end of the 60 min cyanide infusion. To evaluate the metabolic effects of 12.5 mg/kg oxypurinol on purine catabolism in cyanide-poisoned rabbits (*n* = 4), we measured purine metabolites in serial blood samples and then compared them to cyanide-poisoned rabbits that received saline (Table 3 and Figure 3D–I). As expected, oxypurinol significantly decreased the production of end products of purine catabolism: uric acid (−65-fold, *q* = 0.0004) and allantoin (−2.2-fold, *q* = 0.0004). In addition, upstream purine metabolites were significantly increased: inosine (49-fold, *q* = 0.001, Figure 3F), hypoxanthine (20-fold, *q* = 0.001, Figure 3G), xanthosine (2-fold, *q* = 0.016, Figure 3H), and xanthine (4.2-fold, *q* = 0.001, Figure 3I). Taken together, these findings are consistent with enzyme inhibition leading to upstream substrate accumulation (Figure 3J). In sum, in cyanide-poisoned rabbits, oxypurinol treatment inhibited purine catabolism to uric acid and restored tissue cytochrome c oxidase to its reduced state. Thus, in sum, the metabolic and physiological changes associated with cyanide exposure were partially mitigated with a pharmacological inhibitor of xanthine oxidoreductase in an in vivo model.

### 3.4. Uric Acid Correlates with Known Biomarkers of Cyanide Poisoning

**Figure 4:**
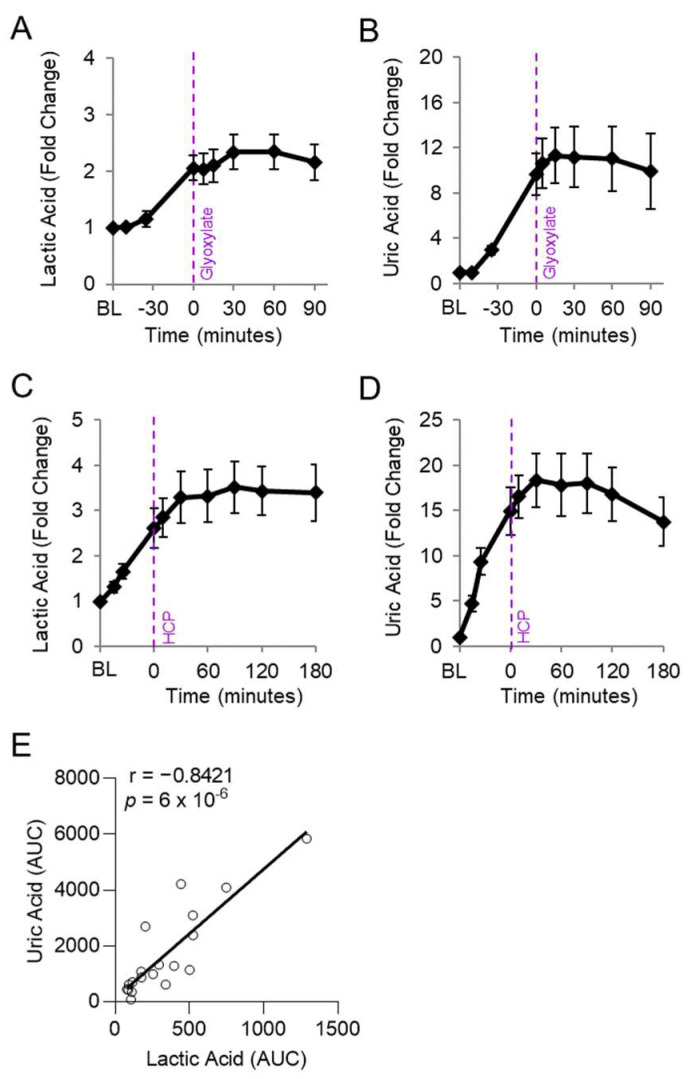
Uric acid correlates with lactic acid in rabbits exposed to cyanide. Rabbits were exposed to a lethal dose of cyanide (t = BL to 0) and treated intramuscularly with an antidote (t = 0): (**A**,**B**) hexachlorplatinate (30 mg/kg; *n* = 9) and (**C**,**D**) glyoxylate (50 mg/kg; *n* = 9). (**A**,**C**) Plasma levels of lactic acid and (**B**,**D**) uric acid are shown in serial samples obtained throughout the study. (**E**) Spearman correlation between the AUCs of lactic acid and uric acid in rabbits treated with either cyanide + glyoxylate or cyanide + hexachloroplatinate.

We assessed whether the abnormally elevated uric acid levels in cyanide-treated animals correlated with lactic acid, a known clinical biomarker of cyanide poisoning, in animals exposed to cyanide and treated with experimental cyanide antidotes. Plasma levels of lactic acid and uric acid were measured in serial samples from rabbits treated with a cyanide-chelating antidote (30 mg/kg IM hexachloroplatinate; *n* = 9) [28] and rabbits treated with a metabolism-based cyanide antidote (50 mg/kg IM glyoxylate; *n* = 9) [24] (Figure 4A–D). As expected, lactic acid levels increased during cyanide exposure and plateaued following treatment with efficacious cyanide antidotes (Figure 4A,C). Similarly, uric acid increased during cyanide exposure and plateaued following treatment with efficacious cyanide antidotes (Figure 4B,D). 

**Figure 5:**
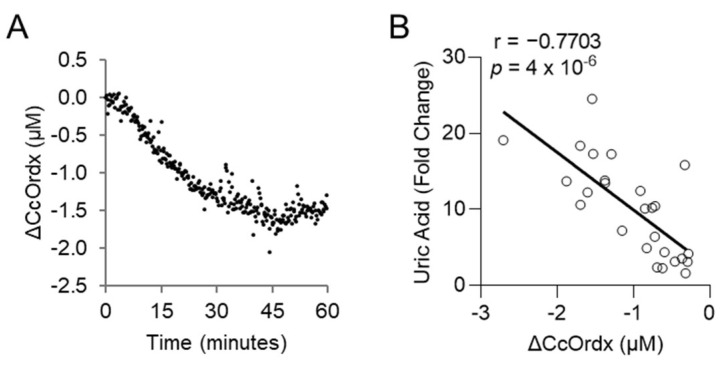
Uric acid correlates with the redox state of cytochrome c oxidase in rabbits exposed to a lethal dose of cyanide. (**A**) Continuous monitoring of cytochrome c oxidase redox state (ΔCcOrdx) in the skeletal muscle of rabbits exposed to cyanide. (**B**) Spearman correlation between the level of plasma uric acid and muscular tissue cytochrome c oxidase redox state (ΔCcOrdx) in cyanide-treated rabbits.

We evaluated whether levels of uric acid statistically correlate with those of lactic acid, an established biomarker of cyanide exposure. For this analysis, the area under the curve for uric acid was compared to that of lactic acid in each animal treated with cyanide + glyoxylate or cyanide + hexachloroplatinate. Uric acid correlated strongly with lactic acid (r = 0.8421, *p* = 6 × 10^−6^; Figure 4E). Finally, we assessed the relationship between uric acid and cytochrome c oxidase redox state. During cyanide poisoning, the cytochrome c oxidase redox state progressively decreased in skeletal muscle, as shown by real-time DOS monitoring (Figure 5A). Plasma levels of uric acid exhibited a strong, inverse correlation with skeletal muscle cytochrome c oxidase redox state (r = −0.7703; *p* = 4 × 10^−6^; Figure 5B). In summary, these findings indicate that uric acid may serve as a potential plasma biomarker of cyanide exposure in addition to a plasma biomarker of cyanide’s effects on skeletal muscle cytochrome c oxidase redox state. 

## 4. Discussion

In this study, we examined the actions of cyanide on purine metabolism in vivo. To accomplish this, we performed targeted metabolite profiling in an established rabbit model of lethal cyanide poisoning. The levels of several concordant metabolites in the purine catabolism pathway were increased in the circulation: uric acid, allantoin, xanthosine, xanthine, hypoxanthine, inosine, and AMP. Further, pharmacological inhibition of xanthine oxidoreductase in rabbits mitigated the metabolic and physiological effects of cyanide. Finally, the end product of purine catabolism, plasma uric acid, strongly correlated with tissue cytochrome c oxidase redox state, in addition to an established plasma biomarker of cyanide exposure, lactic acid.

Under physiological conditions, the concentration of purine metabolites in the plasma is low. In the context of hypoxia and ischemia, there is an increase in purine catabolism within tissues. The resulting products (adenosine, xanthine, hypoxanthine, inosine, and others) are released into the circulation [30,31]. One trigger for the induction of this pathway is ATP depletion. An increase in AMP:ATP ratio activates AMPK which subsequently tries to restore energy balance by inhibiting anabolic pathways and inducing catabolic pathways [32]. AMP is subsequently catabolized through a series of reactions to hypoxanthine/xanthine and ultimately uric acid. Concomitantly, increased hypoxanthine and xanthine reflect a state of ATP depletion [30,33]. While this has been well established in the states of oxygen depletion (hypoxia and ischemia), the extent to which cyanide—an inhibitor of oxygen utilization—affects the purine catabolism pathway is not completely known. Here, we demonstrate elevated levels of concordant purine metabolites in the circulation of cyanide-treated rabbits. This suggests a state of ATP depletion in the cyanide-treated rabbits which is consistent with cyanide’s known inhibition of ATP generation via oxidative phosphorylation. During cyanide exposure, cells shift from oxidative phosphorylation to glycolysis to generate ATP. As the consumption of ATP exceeds the rate of its regeneration, the levels of ATP decrease, and those of ADP and AMP rise. In sum, mass spectrometry-based analysis of purine metabolites showed that the effects of cyanide on purine catabolism in vivo reflect those known to occur in other states of ATP depletion (increased AMP, xanthine, hypoxanthine, and inosine).

XOR, the rate-limiting enzyme is purine catabolism, is broadly expressed and exists in two forms: xanthine dehydrogenase and xanthine oxidase [31]. Intracellularly, XOR exists predominantly in the dehydrogenase form. In certain pathological states, including ischemia and hypoxia, the liver releases the dehydrogenase form into circulation where it is proteolytically cleaved into the oxidase form [30,34,35,36,37]. Each form of the enzyme uses different cofactors; the reductase form of the enzyme uses the cofactors H_2_O + NAD+ and generates NADH + H+, while the oxidase form uses the cofactors H_2_O + O_2_ and generates H_2_O_2_ + O_2_^•−^. Once released into the circulation, the oxidase form of the enzyme binds glycosaminoglycans on the apical surface of the endothelium (*K_d_* = 6 nM) [38,39], damaging vascular beds at multiple sites by consuming xanthine and hypoxanthine and producing superoxide anion and hydrogen peroxide. Presently, in the context of cyanide-induced histotoxic hypoxia, it is not known if the accumulated purine catabolites in the circulation are generated at a tissue site such as the liver or heart or by endothelial-bound XOR that was released into the circulation by the liver or another tissue. Regardless of the source of XOR, XOR inhibitors have the potential to improve tissue function (liver or heart) and improve vascular function during cyanide poisoning.

Interestingly, cyanide anions are known to directly inhibit XOR via multiple mechanisms [17,18,19,20]. The prior in vitro investigations indicate that purine catabolism to uric acid should be inhibited during cyanide exposure in vivo. However, our findings in the rabbit model demonstrate that this is not observed to be the case in a mammalian animal of cyanide exposure, as evidenced by the robust generation of uric acid. This is indirectly consistent with a report by Isom and colleagues; the brains of mice treated with allopurinol and sublethal cyanide exhibited decreased levels of lipid peroxidation [40]. In that study, purine metabolites were not measured. However, blocking XOR with allopurinol was postulated to protect the brain from cyanide by reducing the generation of reactive oxygen species [40].

During cyanide poisoning, cytochrome c oxidase rapidly becomes oxidized. Here, we demonstrated that the XOR inhibitor oxypurinol mitigates the effects of cyanide on the redox status of cytochrome c oxidase in the skeletal muscle of cyanide-treated rabbits. The beneficial effect of oxypurinol was not sufficient to confer survival in rabbits despite inhibiting the cyanide-induced production of uric acid. By contrast, oxypurinol treatment protected zebrafish larvae from dying of cyanide poisoning. This difference may be due to an insufficient dose in the rabbit model or due to differences in drug distribution between the two species. In the tiny zebrafish larvae, the kinetics and concentration of oxypurinol in various tissues may be different as compared to rabbits. Interestingly, a combination treatment of oxypurinol and a known cyanide antidote that acts via chelation enabled a lower antidote dose. This suggests that XOR or purine metabolism plays a role, in part, in the pathogenesis of cyanide toxicity. To confirm this, future studies will assess if XOR inhibitors in combination with cyanide-chelating drugs are efficacious in rabbit or other mammalian models.

In perspective, it is possible that XOR inhibitors can function as a prophylactic medical countermeasure since these drugs were administered before cyanide exposure in rabbits and co-administered with cyanide in the zebrafish. Currently, a prophylactic cyanide countermeasure does not exist. XOR inhibitors may serve this purpose and protect the heart, liver, brain, and vasculature from cyanide-induced damage. This might be of high value in protecting patients treated with nitroprusside to ameliorate the sublethal cyanide exposure that occurs during nitroprusside administration [41]. In addition, these inhibitors potentially may be used to prophylactically treat first responders, firefighters, or soldiers entering known cyanide exposure zones [42,43,44]. Notably, studies conducted on victims of smoke inhalation have demonstrated that blood cyanide levels increase and are associated with increased mortality following smoke inhalation [45]. Therefore, prophylactic anti-cyanide countermeasures will be useful for firefighters chronically exposed to smoke inhalation and soldiers repeatedly exposed to burn pits. Importantly, both allopurinol and febuxostat are FDA-approved medications, with relatively minor side effects, used to treat gout (a condition characterized by hyperuricemia and the formation of uric acid crystals in the joints). However, future studies on patients are required to determine if these medications can improve outcomes in response to acute or chronic cyanide exposure from smoke inhalation, burn pits, and nitroprusside.

There are some limitations to our study. First, we used one dose of oxypurinol which was based on the drug’s solubility limit and the injection volumes allowed in our IACUC-approved protocol. In the future, drug formulation studies are warranted to overcome solubility limits and increase the administered dose. Second, it is not known which tissue(s) are the source of elevated levels of plasma purine metabolites. XOR can be released by the liver into the circulation and generate purine metabolites directly in the blood. Purine catabolic metabolites can also be released into the circulation by the heart and endothelium. In each case, the released XOR can cause local tissue damage or vascular damage, via its effects on metabolism and reactive oxygen species generation. Tissue-specific studies are required to dissect intra- and inter-tissue effects in the context of cyanide. Concordantly, XOR inhibitors could counteract the local effects of cyanide and protect the endothelium and vascular function in a tissue-specific manner. Third, the de novo purine synthetic pathway was not interrogated. De novo purine synthesis may contribute to the observed increase in purine metabolites in rabbits. However, de novo purine synthesis is a highly energy-intensive process. During cyanide exposure, ATP is deleted, thus it is unlikely that flux through the de novo synthesis pathway would be energetically favored during cyanide poisoning.

In sum, despite the essential role of purine metabolites as energy substrates and cofactors, purine metabolism in the context of cyanide is not well understood in living animals. The findings presented here shed new light on the in vivo role(s) of cyanide on purine metabolism. Moreover, there are few clinical biomarkers of cyanide poisoning and a need for additional biomarkers both to assess cyanide intoxication and to evaluate treatment efficacy in preclinical and clinical studies. Plasma uric acid may serve these purposes, functioning both as a plasma marker of cyanide exposure and a surrogate biomarker of skeletal muscle cytochrome c oxidase redox status.

## Figures and Tables

**Table 1:** Plasma TCA cycle metabolite values in rabbits exposed to a lethal dose of cyanide.

Metabolite	Saline (*n* = 5)	Cyanide (*n* = 15)	*p*-Value	*q*-Value
Glucose	1.07 ± 0.05	0.82 ± 0.10	0.0249	0.002802
Pyruvic Acid	1.22 ± 0.22	8.43 ± 1.46	0.0001	0.000028
Lactic Acid	1.16 ± 0.17	3.61 ± 0.95	0.0081	0.001030
Oxaloacetic Acid	0.89 ± 0.03	3.31 ± 0.35	<0.0001	0.000025
Aconitic Acid	0.89 ± 0.05	1.70 ± 0.13	0.0001	0.000028
Alpha-Ketoglutaric Acid	0.78 ± 0.05	3.68 ± 0.55	<0.0001	0.000025
Succinic Acid	0.97 ± 0.14	24.04 ± 4.37	<0.0001	0.000025
Fumaric Acid	1.14 ± 0.14	15.02 ± 2.87	<0.0001	0.000025
Malic Acid	1.07 ± 0.13	4.55 ± 0.61	0.0001	0.000028

Data were calculated from the integrated peak area (counts) using mass spectrometry software, presented as fold change at timepoint 55 min normalized to baseline, and are the mean ± standard error or the mean. *p* values were calculated using Mann–Whitney tests. FDR *q* values were calculated using Benjamini, Krieger, and Yekutieli two-stage step-up method; metabolites with *q* < 0.05 are FDR-significant.

**Table 2:** Plasma purine metabolite values in rabbits exposed to a lethal dose of cyanide.

Metabolite	Saline (*n* = 5)	Cyanide (*n* = 12)	*p*-Value	*q*-Value
Uric Acid	1.28 ± 0.19	16.28 ± 1.27	0.0006	0.00033
Xanthosine	0.69 ± 0.07	3.23 ± 0.19	0.0006	0.00033
Xanthine	0.71 ± 0.08	2.95 ± 0.16	0.0006	0.00033
Hypoxanthine	0.37 ± 0.12	1.79 ± 0.17	0.0013	0.00053
Inosine	0.41 ± 0.10	1.08 ± 0.08	0.0046	0.00157
AMP	1.27 ± 0.50	3.74 ± 0.98	0.0992	0.02863
Adenosine	0.68 ± 0.08	1.72 ± 0.47	0.2544	0.06423
Allantoin	0.73 ± 0.04	1.15 ± 0.03	0.0006	0.00033

Data were calculated from the integrated peak area (counts) using mass spectrometry software, presented as fold change at timepoint 55 min normalized to baseline, and are the mean ± standard error or the mean. *p* values were calculated using Mann–Whitney tests. FDR *q* values were calculated using Benjamini, Krieger, and Yekutieli two-stage step-up method; metabolites with *q* < 0.05 are FDR-significant.

**Table 3:** Effect of oxypurinol on plasma purine metabolite values in rabbits exposed to a lethal dose of cyanide.

Metabolite	Cyanide (*n* = 12)	Cyanide + Oxypurinol (*n* = 4)	*p*-Value	*q*-Value
Uric Acid	16.28 ± 1.27	0.25 ± 0.01	0.0006	0.00040
Xanthosine	3.23 ± 0.19	6.21 ± 1.91	0.0992	0.01670
Xanthine	2.95 ± 0.16	12.52 ± 1.74	0.0006	0.00168
Hypoxanthine	1.79 ± 0.17	37.29 ± 11.19	0.0006	0.00168
Inosine	1.08 ± 0.08	53.71 ± 15.92	0.0006	0.00168
Allantoin	1.15 ± 0.03	0.51 ± 0.01	0.0019	0.00040

Data were calculated from the integrated peak area (counts) using mass spectrometry software, presented as fold change at timepoint 55 min normalized to baseline, and are the mean ± standard error or the mean. *p* values were calculated using Mann–Whitney tests. FDR *q* values were calculated using Benjamini, Krieger, and Yekutieli two-stage step-up method; metabolites with *q* < 0.05 are FDR-significant.

## Data Availability

The data that support the findings of this study are available from the authors on reasonable request. Please contact Anjali K. Nath at anath1@bidmc.harvard.edu.

## Supplementary Materials

The following supporting information can be downloaded at: https://www.mdpi.com/article/10.3390/metabo14050279/s1, Figure S1: Plasma levels of glycolytic and TCA cycle metabolites increase in rabbits exposed to a lethal dose of cyanide; Figure S2: Glycolytic and TCA cycle metabolites increase in zebrafish larvae exposed to a lethal dose of cyanide; Figure S3: Oxypurinol quantification by mass spectrometry; Table S1: Multiple reaction monitoring transitions of compounds and their coefficient of variation in pooled plasma.
