# Supplementary material for: Mass Spectrometric Analysis of Purine Intermediary Metabolism Indicates Cyanide Induces Purine Catabolism in Rabbits
Source: Metabolites. 2024 May 10;14(5):279. doi: 10.3390/metabo14050279 (PMC11123099; doi:10.3390/metabo14050279)
Supplement: Supplementary file 1 [file metabolites-14-00279-s001.zip › metabolites-2992429-supplementary.pdf]

SUPPORTING INFORMATION

Article

Mass Spectrometric Analysis of Purine Intermediary Metabolism Indicates Cyanide Induces Purine Catabolism in Rabbits

Running title: In vivo effects of cyanide on purine metabolism

Jordan Morningstar [1], Jangwoen Lee [2], Sari Mahon [2], Matthew Brenner [2,3] and Anjali K. Nath [1,4,*]

[1] Division of Cardiovascular Medicine, Beth Israel Deaconess Medical Center, Boston, MA 02215, USA; morningj@musc.edu
[2] Beckman Laser Institute, University of California, Irvine, CA 92697, USA; jangwl@hs.uci.edu (J.L.); mahonsb@hs.uci.edu (S.M.); mbrenner@uci.edu (M.B.)
3 Division of Pulmonary and Critical Care Medicine, Department of Medicine, University of California, Irvine, CA 92697, USA; mbrenner@uci.edu (M.B.)
[4] Harvard Medical School, Boston, MA 02215, USA
* Correspondence: anath1@bidmc.harvard.edu

Keywords: mass spectrometry (MS); purine; nucleoside/nucleotide metabolism; metabolic regulation; cytochrome c oxidase (Complex IV); animal model; allopurinol

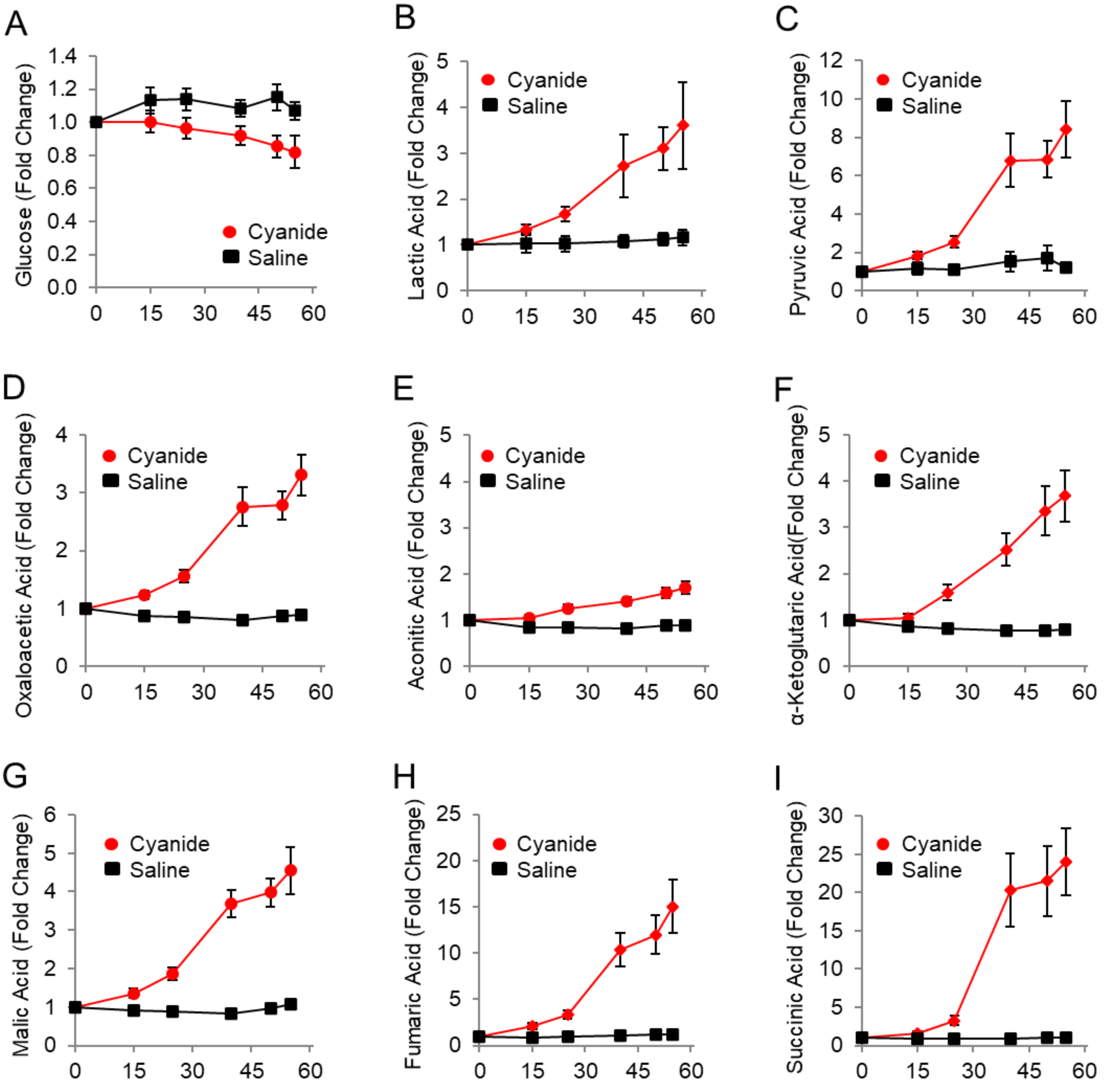

Figure S1. Plasma levels of glycolytic and TCA cycle metabolites increase in rabbits exposed to a lethal dose of cyanide. Metabolites were measured in the plasma of cyanide-treated animals ($n = 15$) and sham control animals ($n = 5$): (**A**) glucose, (**B**) lactic acid, (**C**) pyruvic acid, (**D**) oxaloacetic acid, (**E**) aconitic acid, (**F**) α-ketoglutaric acid, (**G**) malic acid, (**H**) fumaric acid, and (**I**) succinic acid. See Table 1 in the manuscript for P values and q values.

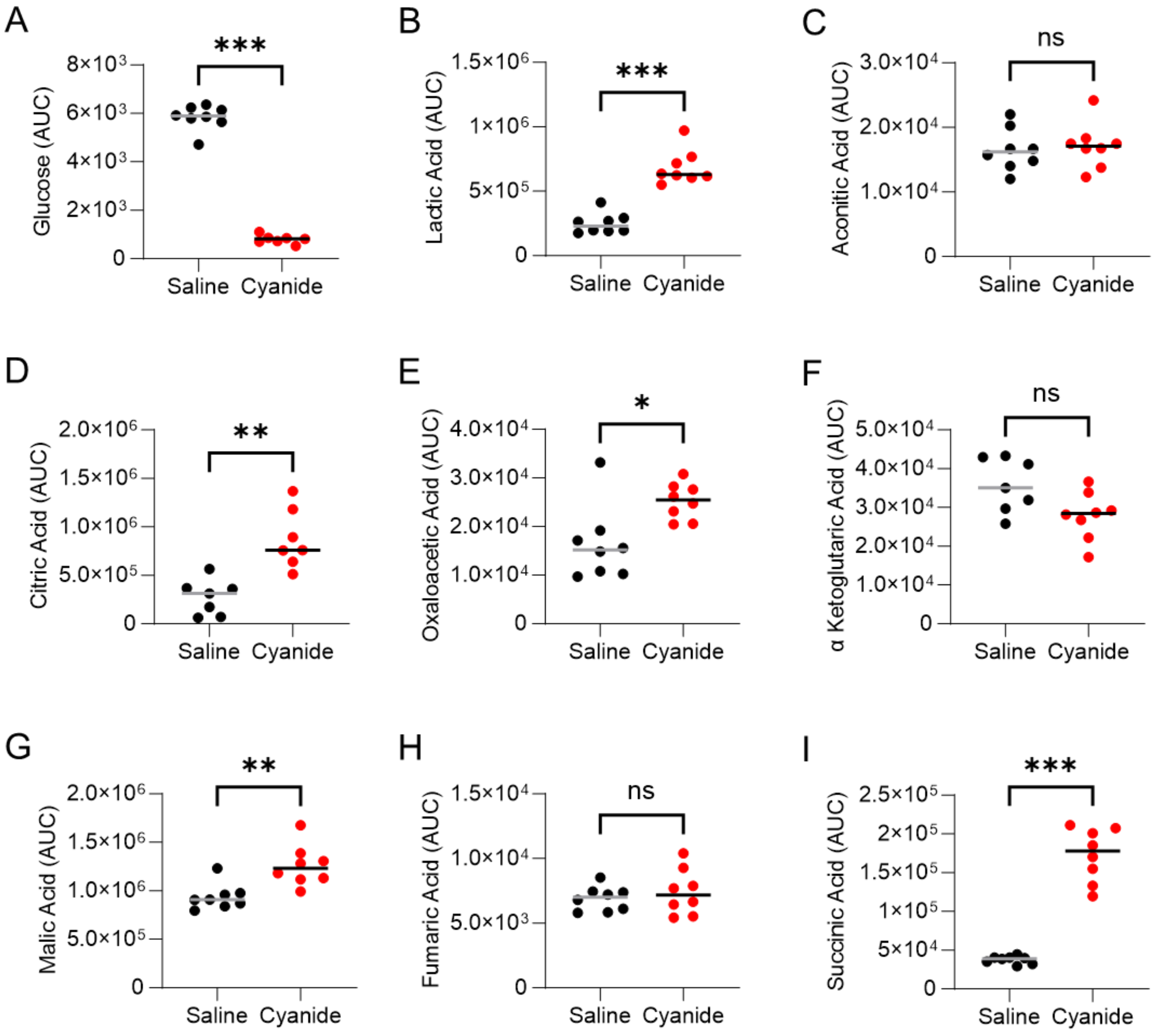

Figure S2. Glycolytic and TCA cycle metabolites increase in zebrafish larvae exposed to a lethal dose of cyanide. Metabolites were measured in lysates (n = 8) from whole zebrafish larvae (6 d.p.f.) exposed to saline or 25 µM potassium cyanide for 2 hours. Each data point represents a group of 10 larvae. (**A**) Glucose (P = 0.00031; q=0.00032), (**B**) lactic acid (P = 0.00015; q = 0.00024), (**C**) aconitic acid (n.s.), (**D**) citric acid (P = 0.00116; q = 0.00091), (**E**) oxaloacetic acid (P = 0.01041; q = 0.00546), (**F**) α-ketoglutaric acid (n.s.), (**G**) malic acid (P = 0.00186; q = 0.00117), (**H**) fumaric acid (n.s.), and (**I**) succinic acid (P = 0.00015; q = 0.00024). ns = P > 0.05; * = P ≤ 0.05; ** = P ≤ 0.01, *** = P ≤ 0.001.

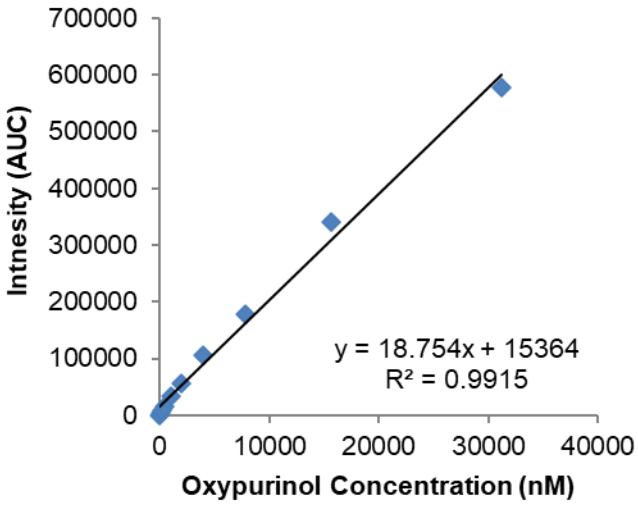

Figure S3. Oxypurinol quantification by mass spectrometry. To generate the oxypurinol standard curve, oxypurinol was spiked into pooled reference plasma, serially diluted, and analyzed by LC-MS/MS. The standard curve was linear from 122 nM to 31.25 µM. The absolute concentration of oxypurinol in treated animals was calculated using this standard curve. See **Table S1** for multiple reaction monitoring (MRM) transitions.

Table S1. Multiple reaction monitoring transitions of compounds and their coefficient of variation in pooled plasma.

Ionization Mode	Q1 Precursor (m/z)	Q3 Product (m/z)	Collision Energy (eV)	Metabolite Name	Coefficient of Variation*
Negative	181.22	138.2	−6	Citrulline-d_7	7.7
Negative	271.2	138.9	−22	Inosine-$^{15}N_4$	8.5
Negative	129.11	42.1	−14	Thymine-d_4	7.2
Negative	172.19	154.1	−6	Phenylalanine-d_8	6.5
Negative	161.1	113.1	−2	Glucose	11.6
Negative	87.05	43	−14	Pyruvic acid	8.1
Negative	89.1	43.1	−16	Lactic acid	5.9
Negative	131.1	87.1	−14	Oxaloacetate	11.3
Negative	191.1	111.1	−15	Citric acid-Isocitric acid	15.0
Negative	173.05	85	−17	Aconitic acid	19.6
Negative	145.1	101.1	−13	α-Ketoglutaric acid	9.1
Negative	117.1	73	−12	Succinic acid	9.1
Negative	115.06	71.01	−13	Fumaric acid	36.8
Negative	133.08	115	−14	Malic acid	18.5
Negative	151.02	42.1	−16	Oxypurinol	7.9
Negative	267.2	135	−27	Inosine	9.9
Negative	346.2	78.8	−50	AMP	23.8
Negative	135.1	92.1	−18	Hypoxanthine	7.6
Negative	157.05	114	−17	Allantoin	16.5
Negative	167.001	124	−17	Uric acid	6.9
Negative	151.1	108	−23	Xanthine	6.4
Negative	283.2	150.9	−24	Xanthosine	21.9

m/z: mass/charge; eV: electron volt. *Calculated from measurements in pooled plasma samples, except for oxypurinol which was calculated from oxypurinol spiked plasma samples.
